# Optical and Electronic NO_x_ Sensors for Applications in Mechatronics

**DOI:** 10.3390/s90503337

**Published:** 2009-05-06

**Authors:** Cinzia Di Franco, Angela Elia, Vincenzo Spagnolo, Gaetano Scamarcio, Pietro Mario Lugarà, Eliana Ieva, Nicola Cioffi, Luisa Torsi, Giovanni Bruno, Maria Losurdo, Michael A. Garcia, Scott D. Wolter, April Brown, Mario Ricco

**Affiliations:** 1 CNR-INFM Regional Laboratory LIT3, via Amendola 173, 70126 Bari, Italy; E-Mails: angela.elia@fisica.uniba.it (A.E.); spagnolo@fisica.uniba.it (V.S.); scamarcio@fisica.uniba.it (G.S.); lugara@fisica.uniba.it (P.M.L.); 2 Physics Department, University of Bari, Via Amendola 173, I-70126 Bari, Italy; 3 Physics Department, Politecnico di Bari, Via Amendola 173, I-70126 Bari, Italy; 4 Chemistry Department, University of Bari, via Orabona 4, 70126, Bari, Italy; E-Mails: ieva@chimica.uniba.it (E.I.); cioffi@chimica.uniba.it (N.C.); torsi@chimica.uniba.it (L.T.); 5 Institute of Inorganic Methodologies and of Plasmas, IMIP-CNR, via Orabona 4, 70126, Bari, Italy; E-Mails: giovanni.bruno@ba.imip.cnr.it (G.B.); maria.losurdo@ba.imip.cnr.it (M.L.); 6 Duke University, Department of Electrical and Computer Engineering, Durham, NC 27708, USA; E-Mails: michael.art.garcia@gmail.com (M.A.G.); woltersd@duke.edu (S.D.W.); abrown@ee.duke.edu (A.B.); 7 ISI Consulting, via Brunelleschi 15, 70010 Casamassima (BA), Italy; E-Mail: mario.ricco@infoisi.it (M.R.)

**Keywords:** Mechatronics, NO_x_, optical sensor, semiconductor based sensor, nanoparticle

## Abstract

Current production and emerging NO_x_ sensors based on optical and nanomaterials technologies are reviewed. In view of their potential applications in mechatronics, we compared the performance of: i) Quantum cascade lasers (QCL) based photoacoustic (PA) systems; ii) gold nanoparticles as catalytically active materials in field-effect transistor (FET) sensors, and iii) functionalized III-V semiconductor based devices. QCL-based PA sensors for NO_x_ show a detection limit in the sub part-per-million range and are characterized by high selectivity and compact set-up. Electrochemically synthesized gold-nanoparticle FET sensors are able to monitor NO_x_ in a concentration range from 50 to 200 parts per million and are suitable for miniaturization. Porphyrin-functionalized III-V semiconductor materials can be used for the fabrication of a reliable NO_x_ sensor platform characterized by high conductivity, corrosion resistance, and strong surface state coupling.

## 1. Introduction

Monitoring and controlling combustion-related emissions are key issues for applications in mechatronics. The exhaust gas from an internal combustion engine, besides water (H_2_O) and carbon dioxide (CO_2_), also contains unburned and partially-oxidized hydrocarbons (HCs), carbon monoxide (CO) and particulate matter (PM). During combustion, the temperature is so high that the endothermic equilibrium between nitrogen and oxygen to form nitrogen oxide (NO) is established, according to the following scheme:

(1) $$\text{Fuel}\left({\text{C}}_{\text{x}}{\text{H}}_{\text{y}}\right)+\text{Air}\to {\text{CO}}_{2}{+\text{H}}_{2}\text{O}+\text{CO}+{\text{SO}}_{\text{x}}{+\text{NO}}_{\text{x}}+\text{PM}+\text{HCs}$$

Subsequent rapid cooling and ejection of combusted gases into the exhaust system freeze this equilibrium, so exhaust gases also contain significant levels of NO, NO_2_ and N_2_O (generally referred to as NO_x_).

Vehicle exhaust emissions are undesirable and can cause serious pollution products due to complex photo-initiated reactions between NO, HCs and O_2_, resulting in the production of ozone and other reactive oxygen species causing the photochemical smog that is plaguing many major cities. Controlling the HCs and NO_x_ vehicles' emissions can have a direct impact on the effective reduction of the urban photochemical smog formation.

**Figure 1.**
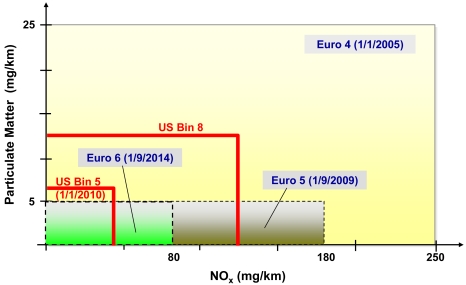
General comparison of emission standards in USA, and Europe.

The US regulations signed in Dec. 2000, introduced very stringent NO_x_ emission standards for all in-use vehicles. In Japan, diesel emission standards require that in-use on-road light commercial vehicles should meet NO_x_ emission of 0.25 g/km starting from the end of 2005 and achieve full implementation by 2011 [1]. The European Commission has also introduced a series of regulations, the so called EURO Emission directives: from Euro I in 1991, to tighter limitations in 2009 (Euro V 80 g/km) and 2014 (Euro VI 0.08 g/Km), to meet the air quality standards stated by the international agencies [2]. Figure 1 illustrates the progression of the Euro and USA emission standard regulations in order to stepwise tighten the limit NO_x_ values.

Extensive research and development efforts have focused on the fabrication of NO_x_ sensors. While the world production of vehicles is presently suffering from the current economic crisis and will presumably reach saturation by 2010, the automotive exhaust gas sensors market is still predicted to grow and reach the level of over 100 million sensors per year in the next few years. High sensitivity, reliability to degradation in harsh environments, and fast response and recovery times are some of the desirable features of such devices. The objective of the present paper is to provide an up-to-date overview of current production and emerging state-of the-art NO_x_ sensors based on optical and nanomaterials technologies. The objective of the present paper is to provide an up-to-date overview of current production and emerging state-of the-art NO_x_ sensors based on optical and nanomaterial technologies.

### 1.1. State of art NO_x_ sensors: achievements and open issues

The key step in controlling car exhaust emissions was the introduction of ‘autocatalysts’ roughly twenty-five years ago. Initially, a platinum-based (Pt) oxidation catalyst was used with an air pump which provided excess air in the exhaust gas to oxidize HCs and CO to less harmful CO_2_ and H_2_O [3]. By the early 1980s, it had been discovered that CO and HCs could be oxidized and NO_x_ reduced simultaneously over a single ‘three-way catalyst’ (TWC) containing Pt and Rh [4]. The correct operating TWC conditions require: i) the total absence of lead in gasoline, which would poison the catalyst; ii) the gaseous mixture has to be set precisely at the stoichiometric value (air/fuel weight ratio equal to 14.6).

The latter requirement can be matched using an oxygen gauge, located in the exhaust gas. The signal delivered by this gauge, also called the lambda gauge, measures the stoichiometry of the emission mixture and determines whether the combustion products are oxygen rich or lean.

Lambda gauges for stoichiometric engines are based on ceramic type metal oxides, usually yttrium stabilized zirconia (YSZ). The YSZ is compacted into a dense ceramic and relies on the generation of mobile oxygen ions (O^2-^) at the elevated temperatures within the tailpipe, typically in excess of 400 °C. The device is fabricated using a pair of high temperature electrodes, such as noble metals (platinum, gold, or palladium) or other metal oxides, is placed onto the surface of the device and an electrical signal, such as the change in voltage or current, is measured as a function of NOx concentration. NO_x_ is reduced at the cathode and the pumped oxygen ion current is a measure for the amount of NO_x_ [5]. The current designs of such NO_x_ sensors are using two pumping cells and chambers. At the first pumping cell, free oxygen is eliminated from the gas by using an oxygen selective electrode. In the second adjacent chamber, NO_x_ is dissociated at a highly catalytic second electrode. The very low amount of NO_x_ (typically < 100 ppm) downstream of a catalyst competes with a high amount of free oxygen. This necessitates careful removal of oxygen from the first chamber without dissociation of NO_x_ at the same time. With high sophisticated electrode materials and a controlled pumping voltage, it is possible to linearly measure 50 ppm NO_x_ in air.

To decrease both fuel consumption and carbon dioxide production and thus contribute to reducing the greenhouse effect, new engines with an excess of air versus the stoichiometric ratio have been developed. As TWC does not operate efficiently when the emission mixture departs from stoichiometry, different solutions have been proposed by car manufacturers [6]. They include either a continuous catalytic reduction of NO_x_ or a chemical trap with periodic regeneration times. Application of a NO_x_ sensor would control the catalyst's operation and monitor the combustion efficiency of the engine. The requirements for such sensors are similar to those of lambda oxygen sensors presently mounted in stoichiometric engines showed that they function reliably, withstand vibrations, are economical, operate at high temperatures, possess low detection limits and are able to operate in harsh environments, such as the corrosive environment within the engine, containing oxygen with water vapor in the range 3 – 8 %.

Current NO_x_ sensor research and development is focused on either optical or electronic methods for detection. NO_x_ optical sensor technology is among the fastest growing for mechatronic applications, as a result of its versatility, ease of use, high speed, accuracy, and capability for integration in high performance automated inspection systems. A wide range of optical sensors based on different operating principles does in fact already exist. Semiconductor laser based sensors are characterized by important properties such as high sensitivity, reliability, possibility of miniaturization, and fabrication that is compatible with mass production. Further development of optical sensors will begin a new era for online inspection of production processes providing the potential for increased productivity and quality. Some key factors still need to be improved in order to reach a wide market, e.g., beam quality, power, wall plug efficiency, wavelength range, tunability, and maximum operating temperature. Electronic metal oxide devices, on the other hand, offer the advantages of low cost, low cross sensitivity to humidity and an output signal which is easy to be read and process. Disadvantages, however, include limitations for operating at high temperatures, signal drift over time, limited selectivity or sensitivity as well as high power consumption. For these reasons, the use of novel materials such as innovative nanostructures, in place of metal-oxides, is being widely investigated in chemiresistors and transistor based device structures. Improvements have been seen for selectivity but operation at high temperature is still an open issue [7-9]. In the following, a brief review of both optical and electronic NO_x_ sensors is reported.

## 2. Optical Sensors

Infrared laser absorption spectroscopy is a powerful tool for sensitive and selective trace gas detection for mechatronic applications. Up to now sensitivities in the range of the parts per million/trillion by volume (ppmv/pptv) have been demonstrated [10].

In the absence of optical saturation and particulate-related scattering, the intensity of light *I*(*x*) propagating in a homogeneous gas of sample length *L* is described by:

(2) $$I\left(\lambda \right)=I_{0}\left(\lambda \right)\exp\left[-L\sigma \left(\lambda \right)c\right]$$

where *I_0_(λ)* represents the initial optical intensity, *I(λ)* the intensity of the radiation after it passes a gas sample of length L where the species to be measured is present at the concentration c, σ(λ) is the absorption cross-section.

In the mid-infrared region of the electromagnetic spectrum (2–14 μm) most molecular species exhibit a unique spectral signature, i.e. a characteristic series of fundamental absorption lines due to transitions between rotational-vibrational states, characterized by very large cross-sections. Hence, mid-infrared spectroscopy is in principle the best choice for the qualitative and quantitative measurements of molecular species in air. Very recently, the development of mid-ir detection techniques have received a significant boost from the invention and development of efficient mid-infrared semiconductor laser sources which promise to substitute optical methods based on the study of overtones and combination of lines falling in the near-infrared spectral region where the absorption cross sections drop by orders of magnitudes.

Among the absorption techniques, direct absorption spectroscopy [10] and cavity enhancement approaches [11] take advantage of long optical path length absorption in multi-pass cells and high finesse optical cavities, respectively. However, in spite of the high sensitivities, these techniques need sophisticated and cumbersome equipments not suitable in mechatronic applications which require compact and transportable sensors.

These limitations can be overcome by fully exploiting the advantages offered by photoacoustic (PA) spectroscopy, which is one of the most effective tools for exhaust gas detection, due to the high sensitivity (parts per billion, ppbv, detection limits), compact set-up, fast response time and portability. PA sensors are based on the generation of an acoustic wave in a specially designed gas cell that arises from the absorption of modulated light of appropriate wavelength by a selected target compound. The amplitude of this sound wave is directly proportional to the gas concentration and can be detected using a sensitive microphone if the laser beam is modulated in the audio frequency range.

In recent years, the development of new mid infrared laser sources has given a new impulse to infrared laser-based trace gas sensors. In particular, single mode quantum cascade lasers (QCLs) have become very attractive for mid-infrared gas sensing techniques thanks to single-frequency operation, narrow linewidth, high powers at mid-IR wavelengths (3 to 24 μm), room temperature and continuous wave (CW) operation [12]. They overcome some of the major drawbacks of other traditional mid-IR laser sources, i.e. lack of continuous wavelength tunability and large size and weight of gas lasers (e.g. CO and CO_2_), large size and cooling requirement of lead salt diode lasers, complexity and low power of nonlinear optical sources.

Recently quantum cascade lasers, working in the 5 – 8 μm range, have been largely used for the spectroscopic detection of NO_x_ with measured high sensitivities in the ppbv range. In the following section, we report on a quantum cascade laser-based photoacustic sensor for the detection of nitric oxide, showing a detection limit of 450 ppbv.

### 2.1. NO photoacoustic sensor

**Figure 2.**
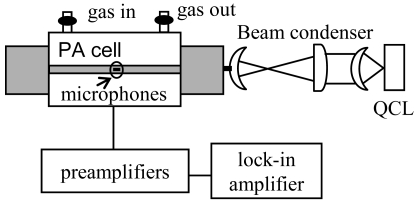
Schematic diagram of the photoacoustic sensor.

The PA sensor consists of a resonant photoacoustic cell, an amplitude modulated single mode QCL and a signal acquisition and processing equipment. Figure 2 shows a schematic diagram of the trace gas sensor.

The resonant PA cell is a cylindrical stainless steel resonator of 120 mm length and 4 mm radius with λ/4 buffer volumes on each side used as acoustic filters for background signal due to the heating of the two ZnSe windows which close the cell at its ends. The resonator is designed to operate in the first longitudinal mode at 1,380 Hz and is equipped with four electret microphones (Knowles EK 3,024, 20 mV/Pa, 0.5 μV/Hz-1), placed on the antinode of the acoustic mode to increase the sensitivity. The electrical signals of the microphones are preamplified and then measured by a digital lock-in amplifier (EG&G Instruments) with a 10 s integration time constant.

The light source is a distributed feedback quantum cascade (QC-DFB) laser, supplied by Alpes Laser (Switzerland) and operated in pulsed mode at a wavelength around 5.3 μm (pulse duration of 42 ns and a duty cycle of 1.4%). The laser is mounted in a Peltier cooled aluminum housing to hold the laser device at a constant temperature chosen between -35 °C and +65 °C. Its light is collected with an AR coated ZnSe lens (2.54 cm focal length, f/1) and collimated by a beam condenser to reduce its diameter in order to avoid the interaction of QCL radiation with the cell walls. The laser beam intensity was modulated by a mechanical chopper at the first longitudinal resonance frequency of the photoacoustic cell. The PA signal was measured by tuning the laser emission over the P(1.5) NO lines located at 1,871.051 – 1,871.066 cm^-1^ and with a maximum intensity of 0.8 × 10^-20^ cm/molecule.

**Figure 3.**
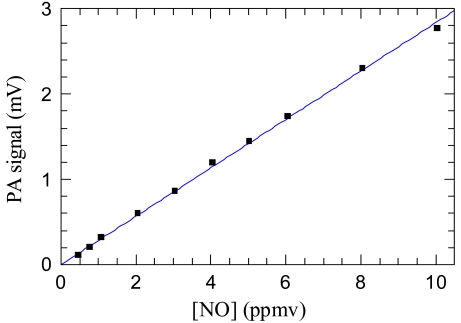
Photoacoustic signal from QCL-based PA cell versus NO concentration.

Figure 3 shows the measured PA signal corrected for in-phase component of the signal, mainly due to the periodical heating of the PA cell windows and walls and measured by filling the PA cell with pure nitrogen. The blue line is a linear fit of the background-corrected data and shows a good linear relationship between the PA signal and the NO concentration. The detection limit *C* for NO diluted in N_2_ was calculated considering a signal-to-noise ratio (*SNR)* of 3 from the following equation:

(3) $$C=\frac{\text{SNR}\cdot \sigma }{a}$$

where *σ* is the standard deviation of the linear fit and *a* the slope of the calibration curve (calibration factor). A detection limit of 450 ppbv was found.

Sensor performance can be described also by the minimum detectable absorption coefficient, normalized to power and detection bandwidth:

(4) $$D=\frac{\alpha_{\min}P_{0}}{\sqrt{\Delta f}}$$

where *α_min_* is the minimum detectable absorption coefficient at a SNR=3, *P_0_* the average laser optical power and Δ*f* the equivalent noise detection bandwidth. In this case, considering *α_min_* = 3.8 × 10^-7^ cm^-1^, *P_0_* = 2 mW and Δ*f* = 0.017 Hz (*τ_int_* = 10 s, 12-dB/octave digital filter) we obtained a detection sensitivity of D = 5.9 × 10^×9^ W·cm^×1^Hz^×1/2^. Table 1 summarizes the state of art PA sensor performances for NO_x_ detection, in terms of detection limit and normalized detection limit. We report the detection limit normalized to the laser optical power (ppbv per Watt), considering that the QCLs optical power has been steadily improved in last few years and now there are commercial sources with optical power up to 80mW [13,14], and prototype laser emitting up to few Watts [15].

## 3. Electronic sensors

### 3.1. Si-Field Effect Gas Sensor

Recent investigations have demonstrated the large potential of monolayer-protected nanoparticles for gas sensing applications [20]. Several studies have been focused on the use of gold-nanoparticle (Au-NP) based gas sensor for the detection of NO_x_. The central thread at the basis is the proven good affinity of NO_x_ with gold surface, as evidenced by Lu and co-workers [21].

Thermally evaporated gold thin film has been used for the monitoring of NO_x_, with little or negligible sensor response towards other interfering gases, such as H_2_ and CO [22]. A straightforward relationship between the grain size of the gold catalytically layer and the sensing response has been envisaged in recent studies, focused on the size-dependent sensor response of Au-NP based sensors [23]. Au-doped micro-porous silicon layers have been employed for NO_x_ monitoring, showing comparable responses toward both NO_2_ and NO species [24]. In 2001, Steffes *et al.* demonstrated the improvement in the sensing behavior of In_2_O_3_ towards NO_2_, after doping with Au-NPs [25].

Subsequently, Langmuir-Schaeffer deposited thiol encapsulated Au-NP films were proposed as sensing material for the detection of NO_2_ down to concentration levels of 0.5 ppm; sensing response was proven to be affected by the chemical composition of the capping molecule and the particle size [26]. Furthermore, Parthangal and co-workers assembled an hybrid system comprising nanostructured gold and zinc oxides nanowires, that allowed the detection of both reducing (methanol) and oxidizing (nitrogen dioxide) gasses at high temperatures [27].

**Figure 4.**
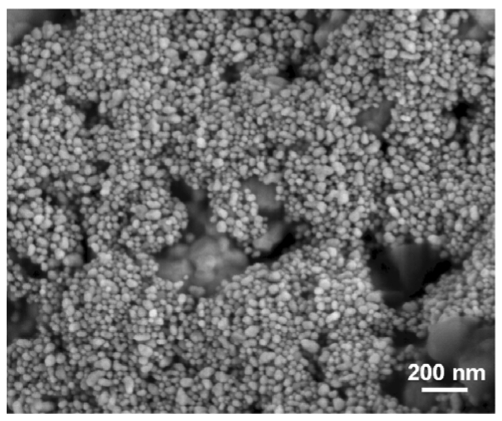
SEM micrograph of thermally annealed Au-NPs. Reprinted with permission from [30].

In our laboratories, we have recently employed electrochemically synthesized Au-NPs as catalytically active materials in field-effect transistor (FET) sensors for NO_x_ monitoring. The synthesis of nanostructured gold was carried out according to the so-called Sacrificial Anode Electrolysis (SAE), first reported in 1994 in a seminal study by M.T. Reetz [28]. Au-NPs with a core-shell structure were electrosynthesized in presence of quaternary ammonium chloride dissolved in THF/acetonitrile mixed solution (mixing ratio 1:3). In this process, the ammonium salt acts as both the supporting electrolyte and the NP stabilizer, forming the particle shell and thus giving rise to a stable Au-NPs colloidal solution. In similar systems, it has been proven that the thickness of the NP outer shell approximately corresponds to the length of the surfactant alkyl chains [29]. Before the use as a gate material in FET sensors, Au-NPs were subjected to a thermal treatment at 200 °C for 1 hour, to increase the nanomaterial conductivity and enhance its stability. The material, after heating, was still nanostructured, with a spherical morphology, although a moderate increase in the NP mean core diameter could be detected (up to 50 nm, see Figure 4 for a Scanning Electron Microscopy micrograph, SEM, of annealed Au-NPs) with respect to the pristine materials (5 nm) [30].

X-ray Photoelectron Spectroscopy (XPS) was used for the surface chemical characterization of both pristine and annealed materials. Carbon (92.3 ± 0.3%_atomic_), nitrogen (3.9 ± 0.3%_atomic_), and chlorine (2.4 ± 0.3%_atomic_), were the most abundant elements detected on the surface of the pristine materials, due to the high amount of surfactant present in the electrolytic environment. Gold (0.1 ± 0.1%_atomic_), and oxygen (1.3 ± 0.3%_atomic_), were present at lower concentration and the carbon to gold elemental ratio was close to 10^3^. After annealing, a higher surface concentration of gold was detected. Indeed, Au, C, N, atomic concentrations were respectively equal to 1.3 ± 0.1%, 34.6 ± 0.3%, 3.2 ± 0.3%, and the C/Au surface atomic ratio was 10^2^. Moreover, the surface of annealed materials showed a significant abundance of signals due to the silicon substrate (Si and O atomic concentrations were equal to 24.7 ± 0.3% and 36.2 ± 0.3%, respectively), which is in agreement with the morphology shown in Figure 4, outlining the presence of holes in the NP active layer, exposing the surface of the underlying SiO_2_ substrate.

**Figure 5.**
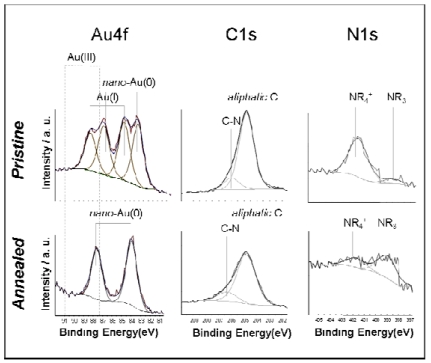
XP spectra and different chemical environments relevant to pristine and annealed Au-NPs. Reprinted with permission from reference [30].

High-resolution XP spectra of pristine and annealed Au-NPs films are shown in Figure 5. The Au4f region of the pristine material (top-left panel) is composed by two doublets, relevant to two chemical states.

The doublet falling at lower Binding Energy (BE; BE_Au4f7/2_ = 83.0 ± 0.1 eV) values is attributed to nano-sized Au(0) [31-33]. The second doublet (BE_Au4f7/2_=84.5 ± 0.1 eV) is attributed to (NR_4_)AuCl_2_ species [34-36]. The relative abundance of these chemical environments showed a certain sample-to-sample variation. After the thermal annealing, the Au4f region changed significantly and only the nano-Au(0) doublet (BE_Au4f7/2_ = 83.7 ± 0.1 eV) was detected. Noteworthy, the slight BE increase observed for this feature is in agreement with the microscopy results, showing a NP size increase upon annealing.

The C1s region of both pristine and annealed nano-films showed the presence of two chemical states, the most abundant one (BE = 284.8 ± 0.1 eV) is due to aliphatic carbon, while the second one (BE = 286.2 ± 0.2 eV) to carbon bound to nitrogen. Both the signals were due to the tetra-alkyl-ammonium salt, used for the Au-NPs preparation. The N1s region was composed by two chemical environments: the first one (BE = 401.8 ± 0.1 eV) is attributed to quaternary nitrogen, while the second one (BE = 399.0 ± 0.2 eV) is attributed to amine species formed by the Hofmann's degradation of the quaternary ammonium during the electrolysis. Sample heating favored further degradation of NR_4_^+^ into lower amines (NR_3_, NHR_2_, etc.), too, and the latter became the main chemical environment in annealed films.

The capacitive FET sensor devices employed consisted of p-doped Si as the semiconductor with a thermally grown SiO_2_ as the insulator. The ohmic backside contact consisted of evaporated, annealed Al. Bonding pads of evaporated Cr/Au were then deposited on the insulator. The sensor chip, a ceramic heater, and a Pt-100 element for temperature control, were mounted on a 16-pin holder and electric contacts made from the sensor to the pins with gold bonding.

**Figure 6.**
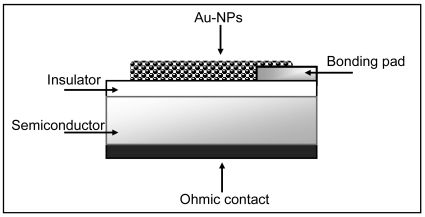
Schematic diagram of the Au-NPs FET sensor.

A fixed volume (0,5 μL) of the colloidal gold solution was drop-cast on the SiO_2_ surface of the capacitor, partially overlapping the bonding pad and subjected to the thermal heating. Figure 6 shows the schematic diagram of the device.

Then, the 16-pin holders were mounted in aluminum blocks connected to a gas line, with a computer-controlled gas mixing system used to regulate the concentration of gases flowed over the sensor surface. Au-NPs-based gas sensors were exposed to NO or NO_2_ gases, in a nitrogen/oxygen carrier gas flow.

**Figure 7.**
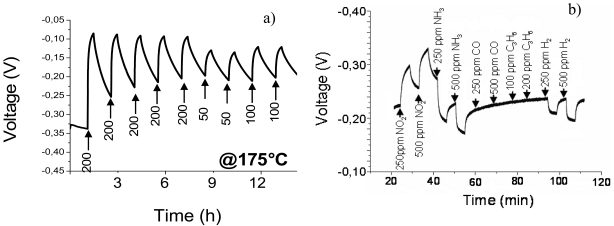
Calibration curve of a Au-NP sensor exposed to NO_2_ in a N_2_/O_2_ carrier flow *(panel b*) and responses of the same sensor to NO_2_ and interfering species *(panel a)*. In both cases the working temperature is 175 °C. Reprinted with permission from reference [30].

A typical calibration curve, recorded in the case of NO_2_ at 175 °C is shown in the panel a) of Figure 7. The sensor showed similar responses in presence of NO. Au-NP sensors were able to detect NO_x_ in a concentration range comprised between 50 and 200 ppm. After the NO_x_ pulse, the sensor was not able to fully recover back to the initial baseline, since some irreversible interaction takes place at the nanoparticle surface between NO_x_ and the nanostructured gold. Similar evidences were reported in several other studies and were explained in light of strong interactions between NO_x_ and gold-based nanoparticles. For instance, these evidences can be found in a study by Filippini *et al.*, who used un-stabilized Au islands [37], and in a paper by Hanwell *et al.*, who used thiol-capped AuNPs [20]. Furthermore, a high response and recovery time was observed by Steffes *et al.* with a working temperature below 350 °C in a gold-modified In_2_O_3_ film [38]; similar results were also reported by Penza *et al.* with WO_3_ thin films activated by gold [39].

Moreover, Vitale *et al.* found spectroscopic evidences of NO_x_ coordination to organometallic Pd^(II)^/thiol-gold nanoparticles hybrids [40], while Koel studied the absorption or bonding geometries of N_x_O_y_ species, i.e., nitrogen dioxide (NO_2_), dinitrogen trioxide (N_2_O_3_), and dinitrogen tetroxide (N_2_O_4_), on Au(111) by means of infrared reflection-absorption spectroscopy [41].

Finally, in the above-cited work of Lu and al. a theoretical investigation by means of ab-initio and density functional calculations supported the stability of several gold-NO_2_ complexes is also reported. [21].

In the panel b) of Figure 7, a typical experiment aiming to quantify the NO_2_/interferent response ratio is reported. The sensor was exposed to different gases: CO, H_2_, NH_3_, C_3_H_6_ and NO_2_.

It is noteworthy that the system used in Figure 7, although less sensitive to NO_x_ than other ones (see for details Table 2, summarizing the state of art Au-based sensor performances for NO_x_ detection, in terms of detection limit), proved to be extremely selective, as no response at all was detected in case of CO, and hydrocarbons. Small signals were recorded in case of NH_3_ and H_2_.

## 4. III-V Semiconductor Based Sensor

III-V compound semiconductors, such as GaAs, have been chemically functionalized with porphyirins molecules and proposed as a method of for selective detection of NO [42]. Among the various porphyrin compounds, hemin (Fe-porphyrin) has been shown to be ideal for sensing NO due to the strong binding affinity it has to this small molecule [43,44]. Generally, there are a host of porphyrin compounds available with distinctly unique properties, and specificity to various gas molecules, that can be exploited for sensor applications. Of scientific interest is the integration of an organic compound, such as the porphyrin molecules, with an inorganic material and the capability for engineered sensor attributes.

The III-V compounds are especially interesting as transduction platforms for reasons of chemical inertness and wide ranging electronic properties. Detection of NO by way of hemin functionalized to GaAs has been shown to decrease its surface potential, which caused an increase in current throughout the GaAs device [45]. As such, another favorable property of these semiconductors is its sensitivity to adsorbates.

In recent years, the authors have evaluated InAs and AlGaN/GaN HFET structures functionalized with hemin to study chemical bonding state and associated sensor function when exposed to various analytes including NO. The important aspect of n-InAs is its characteristic surface accumulation layer which is modified based on changes in surfaces states due to adsorption of analyte molecules. Similarly, the buried 2-dimensional electron gas in AlGaN/GaN heterojunction field effect transistor (HFET) structures is also coupled to surface charge states and sensitive to modifications in charge carrier concentration upon analyte capture. We have used surface-sensitive techniques in our work, such as spectroscopic ellipsometry, to determine the thickness and/or coverage of the functionalizing layer; x-ray photoelectron spectroscopy, to reveal the hemin/semiconductor binding chemistry; and atomic force microscopy (AFM) to evaluate surface topology of the III-V compounds functionalized by hemin [46]. Herein, we present an overview of surface chemistry and sensor function results for hemin functionalized surfaces and its response to NO. The approach we devised for obtaining electrical data for NO gas detection is based on the established four-contact van der Pauw (VDP) method for characterizing sheet resistivity [47, 48]. This methodology differs from current chemical sensors in a variety of ways. One such differentiation is the enhanced accuracy of the four point probe averaging technique reducing the effects due to contact resistance. Another benefit is the relative simplicity of the device and ease of manufacture.

**Figure 8.**
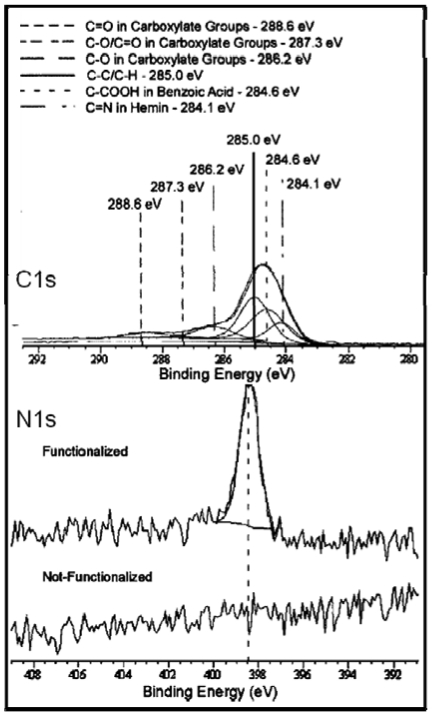
XPS data with deconvoluted Gaussian-Lorentzian component fits of an InAs surface in the C 1*s* region and before and after functionalization with hemin (1mM, 3h dipping) in the N 1*s* region.

**Figure 9.**
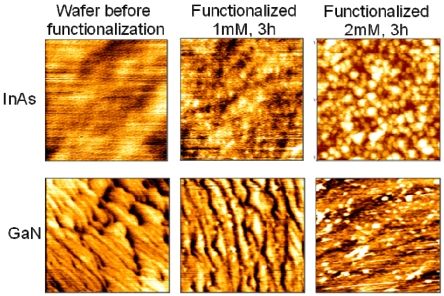
500 nm × 500 nm AFM images of InAs and GaN surfaces before and after functionalizeation with 1 and 2 mM hemin solutions. White dots for 2mM solution indicate hemin aggregates formation.

Hemin chloride was acquired from Frontier Scientific, Inc. and 1–2 mM concentrations in dimethylformamide were used. InAs (001) and AlGaN/GaN(0001) surfaces were immersed into the hemin solution for 1–3 h. The samples were subsequently rinsed with a 5% by volume chloroform/hexane solution and dried with N_2_ gas. The goal of this procedure was to obtain a uniform monolayer of dispersed hemin molecules chemically attached onto the sample surface. The presence and efficacy of the procedure was evaluated by XPS and some typical data are seen in Figure 8. One notable feature of the C 1s core level XPS scan is a peak at ∼ 284 eV observed for the functionalization chemistries, which is attributed to C=N bonding within the porphyrin molecule [49]. Furthermore, for hemin-functionalized InAs surfaces, a strong N1s peak from the hemin molecule seen for the functionalized surface provides evidence for the semiconductor hemin functionalization. Interestingly, this work also revealed chemical bonding of the carboxylic acid ligands on the hemin to the anion of the semiconductor. The AFM images of InAs and GaN functionalized with hemin are shown in Fig. 9. The 500×500 nm2 images show the appearance of grain-like features whose size and density increases with the increase of hemin concentration from 1 to 2 mM. Those features are due to hemin aggregates [46], which are inactive from the sensing perspective and therefore, their formation has to be avoided. AFM and ellipsometry data have been used to perfect the functionalization chemistry procedure and determined an optimal concentration <1mM to yield homogeneous porphyrinated monolayers with a thickness of 17 Å [42].

**Figure 10.**
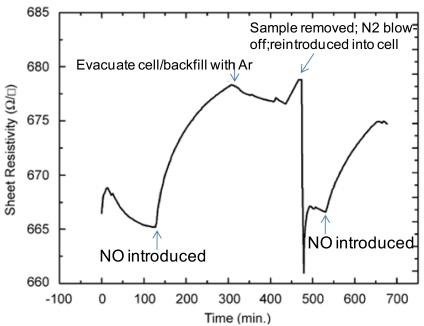
Sheet resistivity of the hemin functionalized GaN HFET as a function of NO exposure.

**Figure 11.**
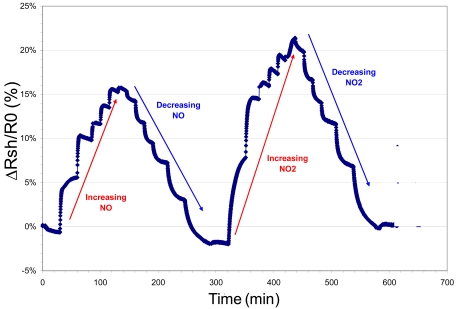
VDP data from a hemin functionalized InAs sample.

The sheet resistivity response of a hemin functionalized AlGaN/GaN HFET structure is shown in Figure 10. These early, initial results show that the functionalized samples show significant changes in sheet resistivity when exposed to 70 ppm of NO in argon. The plot shows attempts to purge the NO gas from the sensor environment for evaluating reversibility and response times. Upon exposure to NO there is a response with an easily measured change in sheet resistivity. The removal of the sample from the sample cell, exposure to dry nitrogen and then reinsertion into the cell did decrease the sheet resistivity to near its initial value. The re-exposure of the structure to NO again changed the sheet resistivity to near its previous NO exposed value. The resistivity response of functionalized InAs to NO and NO_2_ is shown in Figure 11 in terms of the normalized sheet resistivity response ((Rsh – R_0_)/R_0_). The graphic depicts increasing levels of both gases exposed to the sensor followed by decreasing levels of NO and NO_2_ (10 to 80 ppm).

The resistivity response of porphyrinated InAs to NO and NO_2_ generally shows an exponential increase with analyte exposure and decrease with diminishing concentrations, eventually reaching a constant level. This exponential behavior is attributed to Langmuir adsorption kinetics common in semiconductors with responsive surface space-charge regions [52]. Adsorption of NO by the porphyrin molecule tethered to the III-V semiconductor causes conductivity changes through polarization induced bond formation or through van der Waals interaction leading to valence band-bending and/or charge transfer. The InAs surface accumulation layer, with its Fermi level above the conduction band minimum (CBM) balances its highly mobile free-electron concentration with positive surface states [53]. During the adsorption of NO and NO_2_, the space charge region at the InAs surface is affected and has a direct impact on the conductivity change because as the electrons are depleted from the accumulation layer [54].

## 5. Conclusions

Strict emission regulations and deeper environmental awareness have led to intense research into emissions reduction by various engine manufacturers and research institutes. Under these circumstances, NO_x_ detection is becoming a key aspect, and several sensing and transduction mechanisms have been investigated in the recent literature. In the present study, quantum cascade laser based photoacoustic (QCL-PA) detectors, gold nanoparticle-based field-effect transistor sensors (Au-NP-FET) and functionalized III-V semiconductor based devices have been developed, characterized and explored, in terms of sensitivity and selectivity. In Table 3 the sensing properties of the reported NO_x_ sensors have been summarized and compared with commercial YSZ lambda gauges.

The QCL-based PA sensor consists of an amplitude modulated QCL, a PA cell, and a signal acquisition and processing equipment. A resonant configuration of the PA cell, excited in its first longitudinal mode, has been chosen in order to increase the sensitivity, with a detection limit of 450 ppb (SNR =3). The actual device scheme makes the reported PA sensor more suitable for stationary exhaust gas testing than for on-line applications. Future developments will be the implementation in the device scheme of: i) a QC laser operating at room temperature in continuous mode, in order to avoid the thermal chirping of pulsed operation, thus increasing the selectivity; ii) optical microphone, characterized by high sensibility (5 V/Pa) to minimize the electromagnetic noise; iii) fiber coupling with collimating system between the QC laser and the PA cell to take rid of any optical alignment issue and thus obtaining a more compact, robust and portable PA sensor.

Metal Insulator Semiconductor field effect devices used as gas sensors can be of different types: transistors, Schottky diodes or capacitors, with transistors being preferred for commercial devices. The gas sensing principle for field effect sensors is based on molecules adsorbing and dissociating on a catalytically active gate material on the sensor. These interactions create a change in the electric charges on the semiconductor surface, which in turn results in a shift in the sensor output voltage.

The interactions of the gas molecules with the gate material depend on the operating temperature and the morphology and chemical characteristics of the gate material. Electrochemically synthesized gold-nanoparticles, were used as catalytically active materials in field-effect transistor (FET) sensors capable to monitor NO_x_ in the concentration range comprised between 50 and 200 parts per million. The use of nanostructured films as gate material has the potential to give sensors with increased sensitivity, and faster response and recovery due to the larger surface area available for interaction with the gas molecules, as compared to conventional thin film sensing layers. These sensors displayed an appreciable selectivity towards NO_x_, providing small responses to NH_3_ and H_2_ and not responding at all to CO and C_3_H_6_.

Reliable NO sensor could be fabricated with a porphyrin as the functional group and a GaN-based HFET as the semiconductor material. Among the various porphirins, hemin was proposed for the detection of NO because of its chemical affinity, affording for a preferential binding to NO, when exposed to a mixture of gases such as NO, O_2_, and CO. The reported sensing results represent initial data for porphyrinated III-V semiconductor based sensors and show (i) unambiguous response to NO and NO_2_, (ii) proof of Langmuir –type adsorption operative in the sensor function, and (iii) the reversibility and sensitivity of the sensors. Analyte selectivity experiments are underway, but our proof-of-concept devices showed obvious selectivity to NO,compared to O_2_, CO_2_, N_2_, using hemin porphyrin. We have to underlined that InAs and AlGaN/GaN porphyrins functionalized sensors represent completely new systems, that is no previous study on this type of systems have been reported so far. Only GaAs functionalized with hemin based sensor have been reported in literatore as NO sensor. And even in that case the system was neither optimized from functionalization or mechanism point of views.

Further studies are in progress for the optimization of the InAs and AlGaN/GaN porphyrins sensors with respect to sensitivity and selectivity. Nevertheless, since their novelty and promising results they are worth of consideration.

## Figures and Tables

**Table 1.** State of the art of optical spectroscopic detection of NO_x_ by compact PA spectroscopy.

**Reference**	**Chemicals**	**Laser source**	**Detection limit at SNR = 3 (ppbv)**	**Normalized detection limit (ppbv · W)**
Elia *et al.* [16]	NO	Pulsed QCL5.3 μm Room temperature	450	0.9
Kosterev *et al.* [17]	N_2_O	cw QCL4.55 μm Liquid N_2_ cooled	12	1.2
Lima *et al.* [18]	N_2_O, NO_2_	Pulsed QCLs6.2 μm; 8 μm Room temperature	240	1.2
Pushkarsky *et al.* [19]	NO_2_	cw QCL (external grating cavity)6.3 μm Room temperature	1.5	0.45

**Table 2.** State of art Au-NPs based sensor performances for NO_x_ detection.

**Reference**	**Chemicals**	**Catalytically active material**	**Detection limit**
Ieva *et al.* [11]	NO, NO_2_	Core-shell Au-NPs stabilized by tetraalkylammonium chloride	50 ppm @175 °C
Hanwell *et al.* [20]	NO_2_	Core-shell Au-NPs functionalised by 4-methylbenzenethiol, 1-hexanethiol or 1-dodecanethiol	0.5 ppm @22 °C
Filippini et al [22] D. Filippini, L. Fraigi, R. Aragon, U.Weimar, Thick film Au-gate field-effect devices sensitive to NO2, Sensors and Actuators B 81 (2002) 296-300.	NO_2_	Thermally evaporated gold thin film	15 ppm @180 °C
Baratto *et al.* [23]	NO, NO_2_	Au-doped micro-porous silicon layers	5 ppm @20 °C
Steffes *et al.* [24]	NO_2_	Au-NPs modified RF-sputtered In_2_O_3_ film	10 ppm @400 °C

**Table 3.** Detection limit and selectivity values of QCL-based PA sensors, Au-NPs FETs, Hemin-GaN HFETs and commercial YSZ lambda gauges.

	**Detection limit (ppm)**	**Selectivity**
QCL-based PA sensor	0.45	HC, CO_2_, H_2_O, NO_2_, N_2_O, SO_x_
Au-NPs FET	50	NH_3_, H_2_, CO and C_3_H_6_.
Hemin-GaN HFET	4	O_2_, CO_2_, N_2_
YSZ lambda gauges	50	low
